# Novel scoring system provides high separation of diploidy and triploidy to aid partial hydatidiform mole diagnosis: an adaption of HER2 D-DISH for ploidy analysis

**DOI:** 10.1136/jcp-2023-209265

**Published:** 2024-03-30

**Authors:** Caroline M Joyce, Susan Dineen, Julie Deane, Niamh Conlon, Paula M O'Shea, Paul Corcoran, John Coulter, Keelin O'Donoghue, Brendan Fitzgerald

**Affiliations:** 1 Pregnancy Loss Research Group, Department of Obstetrics and Gynaecology, University College Cork, Cork, Ireland; 2 Bichemistry & Cell Biology, University College Cork, Cork, Ireland; 3 Department of Pathology, Cork University Hospital, Cork, Ireland; 4 Department of Biochemistry & Diagnostic Endocrinology, Mater Misericordiae University Hospital, Dublin, Ireland; 5 National Perinatal Epidemiology Centre, University College Cork, Cork, Ireland; 6 Department of Obstetrics & Gynaecology, Cork University Maternity Hospital, Cork, Ireland

**Keywords:** IMMUNOHISTOCHEMISTRY, Classification, Pathology, Molecular

## Abstract

**Aims:**

Diagnosis of hydatidiform mole or molar pregnancy based on morphology alone can be challenging, particularly in early gestation, necessitating the use of ancillary techniques for accurate diagnosis. We sought to adapt the VENTANA *HER2* dual-colour dual-hapten in-situ hybridisation (D-DISH) assay by using the internal chromosome 17 enumeration probe to determine ploidy status.

**Methods:**

We selected 25 products of conception, consisting of molar and non-molar cases, to validate the *HER2* D-DISH assay. These cases had prior morphological assessment by a perinatal pathologist and ploidy analysis using molecular cytogenetics. Three independent observers, blinded to the original histopathological and genetic diagnosis, scored 10 representative areas on each slide. Interobserver variability was assessed by comparing the total scores of each observer using analysis of variance (ANOVA) and the kappa statistic.

**Results:**

Our ploidy scoring system accurately determined the correct number of diploid and triploid conceptuses, demonstrating complete concordance with pre-existing ploidy status and the initial diagnosis. Interobserver agreement between three independent scorers was robust: ANOVA (p=0.36) and kappa statistic (0.812, p<0.001). We achieved clear separation of average nuclear signals for diploid and triploid conceptuses, which was statistically significant (p<0.05). Employing our innovative scoring system, known as the ‘rule of 5’, we established ploidy decision thresholds for all 25 cases.

**Conclusions:**

Our modified *HER2* D-DISH ploidy assay simplifies the process of ploidy determination and improves the accuracy of morphological diagnosis of molar pregnancy. The *HER2* D-DISH assay was selected for ploidy analysis due to the widespread availability of in-situ hybridisation in pathology laboratories.

**WHAT IS ALREADY KNOWN ON THIS TOPIC**

- The *HER2* D-DISH (dual-colour dual-hapten in-situ hybridisation) assay is used extensively in pathology laboratories to determine *HER2* amplification status, primarily in breast and gastric cancers.

**WHAT THIS STUDY ADDS**

- This study demonstrates how an adaption of the *HER2* D-DISH assay, which focuses on nuclei with the highest numbers of nuclear signals from chromosome 17 centromeric probes, facilitates ploidy assessment. This adapted *HER2* D-DISH assay offers an accurate, reliable adjunct to morphology for partial hydatidiform mole diagnosis.

**HOW THIS STUDY MIGHT AFFECT RESEARCH, PRACTICE OR POLICY**

- The *HER2* D-DISH ploidy scoring system achieves high separation between diploidy and triploidy, helping pathologists achieve more accurate hydatidiform mole diagnosis. This may help decrease diagnostic errors, reduce patient anxiety and lower human chorionic gonadotropin surveillance costs. Furthermore, it may facilitate the collection of more reliable incidence data, influencing future research, clinical practice and service development.

## Introduction

 Gestational trophoblastic disease (GTD) covers a wide spectrum of disorders from the pre-malignant conditions of hydatidiform mole (HM) to the malignant disorders of choriocarcinoma, invasive HM, placental site trophoblastic tumour and epithelioid trophoblastic tumour. HMs may be further classified into complete (CHM) or partial (PHM) based on their characteristic morphological features and genome complement.^1^ CHMs mostly have a diploid (2n) androgenetic genome, whereas PHMs mostly have a triploid (3n) diandric monogynic genome.

Diagnosis of PHM and CHM can often be made on morphology alone when the characteristic morphological features of HM are present. It is important to accurately classify HMs as the risk of progression to gestational trophoblastic neoplasia (GTN) is lower for PHM (0.5–1%) than for CHM (13–16%).^2^ However, there are some reported cases of PHM progressing to GTN.^3^,^5^

Advances in ultrasonography have resulted in earlier detection of HMs when typical morphological features may be subtle or absent, making the pathological diagnosis more challenging.^6^ Early pregnancy loss tissue specimens may exhibit atypical villous morphology, sometimes due to aneuploidy, which can mimic HM, making it difficult to distinguish PHM from hydropic non-molar miscarriage.^7 8^ Diagnosis of PHM on morphology alone has an error rate of at least 20%.^9^ Consequently, ancillary techniques have diagnostic utility in differential diagnosis when morphology is subtle or atypical and can help distinguish triploid PHM from diploid gestations. This can allow women with confirmed non-molar miscarriage avoid human chorionic gonadotropin (hCG) monitoring and attempt a new pregnancy without delay.

p57 immunohistochemistry is frequently used as a diagnostic tool to confirm CHMs as in these cases, abnormal absence of staining for p57 in stromal and cytotrophoblast cell nuclei supports the diagnosis.^10^ When p57 staining is normal (ie, cytotrophoblast and stromal nuclear staining is maintained), and/or where morphology assessment raises a differential diagnosis of PHM, ploidy analysis can identify a diploid or triploid conceptus and help distinguish PHM from non-molar miscarriage. Ploidy analysis can be determined using flow cytometry, fluorescent in-situ hybridisation or chromogenic in-situ hybridisation (CISH). Galea and colleagues have used silver in-situ hybridisation (SISH) for the determination of ploidy in suspected molar pregnancy and others have adapted *HER2* CISH assays to determine DNA ploidy.^11^,^13^ The *HER2* dual-colour dual-hapten in-situ hybridisation (D-DISH) assay is readily available in many pathology laboratories for determining *HER2* gene amplification status in women with breast cancer. This assay incorporates a chromosome 17 enumeration probe (CEP17) which can be used to provide ploidy status.

Molecular genotyping can infer ploidy while also elucidating the genetic origin of the additional chromosomal complement in the triploid conceptus. It can also inform the differential diagnosis of a twin pregnancy with HM, mosaics and discordant p57 immunohistochemistry.^814^,^16^ Furthermore, it can help identify familial recurrent HM, a rare autosomal recessive disorder due to pathogenic variants in various genes (*NLRP7*, *KHDC3L* and *PAD16*).^17^,^19^

Triploidy is a common chromosome anomaly occurring in 1–2% of all conceptions.^20 21^ In our clinical practice, we routinely send samples of all placentas from second trimester pregnancy loss and selected recurrent first trimester pregnancy loss products of conception (POCs), for genetic analysis to identify aneuploidies and genomic imbalances. The impetus for this study arose from an incidental finding of triploidy in some POCs following cytogenetic analysis, where subtle morphological features of PHM had led to the initial underdiagnosis of PHM. This led to the search for an ancillary technique that would be accessible in our pathology laboratory to aid diagnosis in POCs with features suspicious for PHM.

In this study, we sought to adapt the VENTANA *HER2* D-DISH assay by using the internal CEP17 probe for ploidy analysis.

## Materials and methods

### Study design

25 atypical POCs, whose cytogenetic results were available, were randomly selected for verification of the *HER2* D-DISH assay. These POCs consisted of a mixture of CHMs, PHMs and non-molar pregnancies. All cases had morphological assessment by a perinatal pathologist and confirmation of ploidy status by prior genetic analysis. The *HER2* D-DISH assay was adapted for ploidy analysis by using the internal CEP17 probe to determine ploidy status. Misinterpreting trisomy 17 as triploidy was considered and deemed highly improbable given the rarity of trisomy 17 in pregnancy loss specimens (1 in 1000 miscarriages).^22 23^

### Confirmatory genetic analysis

We send POCs from selected first and second trimester pregnancy loss to an external laboratory for comprehensive genetic analysis. During this process, evaluation of aneuploidy was conducted by quantitative fluorescent PCR targeting chromosomes 13, 15, 16, 18, 21 and 22, and the sex chromosomes. Genotyping of 42 polymorphic genetic markers known as short tandem repeats and 2 non-polymorphic sex chromosome markers, namely SRY and amelogenin, was performed using the Devyser Extend kit V.2 (Devyser, Sweden). Genomic imbalances, including deletions and duplication, were detected through multiplex ligation-dependent probe amplification analysis, using the subtelomeric kit SALSA P036-E1 (MRC Holland).

### Chromosome 17 *HER2* D-DISH analysis

**Figure 1:**
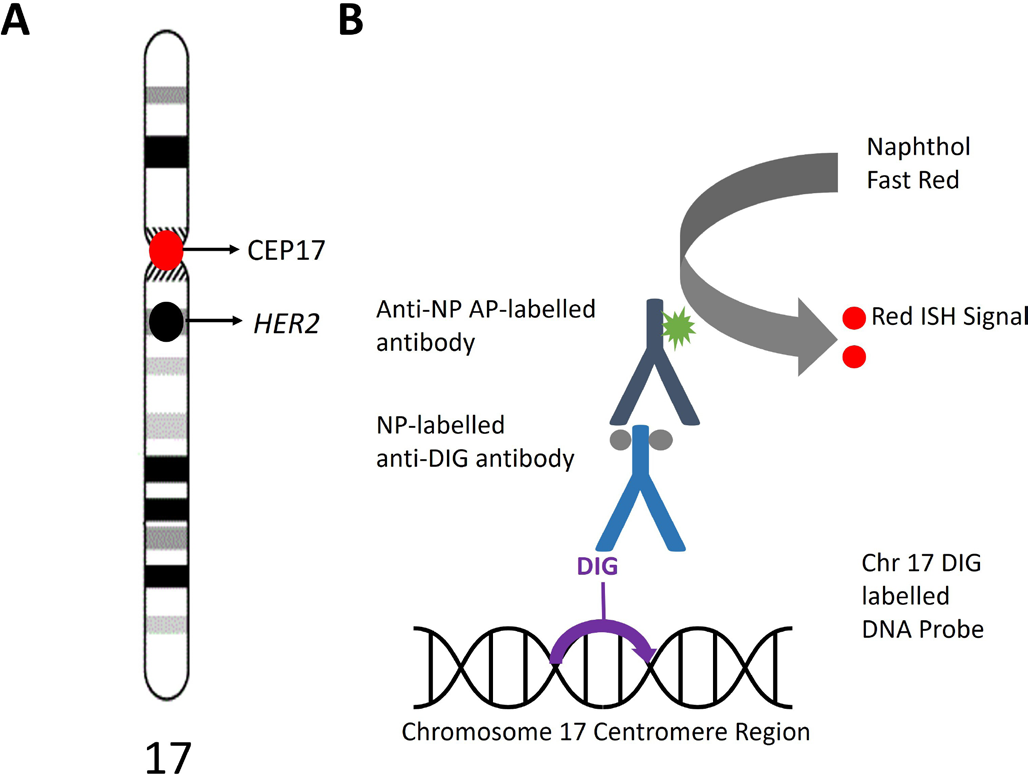
Red in-situ hybridisation (ISH) digoxigenin (DIG)-labelled chromosome 17 detection system. (A) Ideogram of chromosome 17 showing the HER2 locus and chromosome 17 centromeric locus which binds the enumeration probe (CEP17). (B) Illustration of the red ISH DIG detection system. A chromosome 17 DNA probe labelled with DIG targets the centromeric region to provide information on ploidy. An anti-DIG antibody labelled with nitropyrazole (NP) is detected by a secondary antibody conjugated with alkaline phosphatase (AP) which cleaves the substrate (Naphthol Fast Red) to produce a red signal. Adapted from the Roche VENTANA HER2 Dual ISH Method Sheet (2020-07-27, Rev A).

The VENTANA *HER2* D-DISH assay was performed using 4 µm tissue sections from selected formalin-fixed paraffin-embedded (FFPE) blocks derived from the 25 POC cases. This analysis was performed on the Benchmark Ultra-Advanced automated staining system (Roche Diagnostics). The *HER2* D-DISH assay is a closed system incorporating two DNA probes, each employing distinct chromogenic detection systems. The SISH dinitrophenyl probe detects *HER*2, while the red in-situ hybridisation digoxigenin probe binds to the centromeric region of chromosome 17, as illustrated in figure 1. The procedure was performed according to manufacturer instructions and slides were counterstained with haematoxylin for interpretation by conventional bright-field light microscopy.^24^ The *HER2* D-DISH-stained slides were reviewed for staining adequacy and the staining quality was monitored by internal quality assessment. The study was performed in a pathology laboratory accredited to ISO15189 international standards which participates in external quality assurance for *HER2* and in-situ hybridisation (UKNEQAS and Nordic).

A ploidy score was derived by counting the red in-situ hybridisation signals within the nucleus of villous stromal cells. For the nuclei to be eligible for scoring, they had to meet specific criteria: nuclei must not overlap, and red signals must be contained within the nucleus and must be separate from one another. During the signal counting process, we deliberately chose villi that met certain criteria: villi were selected to avoid excessive cellularity that could lead to nuclei overlapping, and highly hydropic villi were excluded as the stroma would be too paucicellular. Consequently, villi with an intermediate level of cellularity were selected for assessment in each POC.

Scoring was performed by three independent observers: a perinatal pathologist, a gynaecological pathologist and a medical scientist. An average of the three scores was taken for each case. All observers were blinded to the original histological and genetic diagnosis. Before formal scoring, a preliminary evaluation of the slides was performed to identify areas of the slide with the cleanest CEP17 signals to facilitate accurate analysis.

### Development of a ploidy scoring system

**Figure 2:**
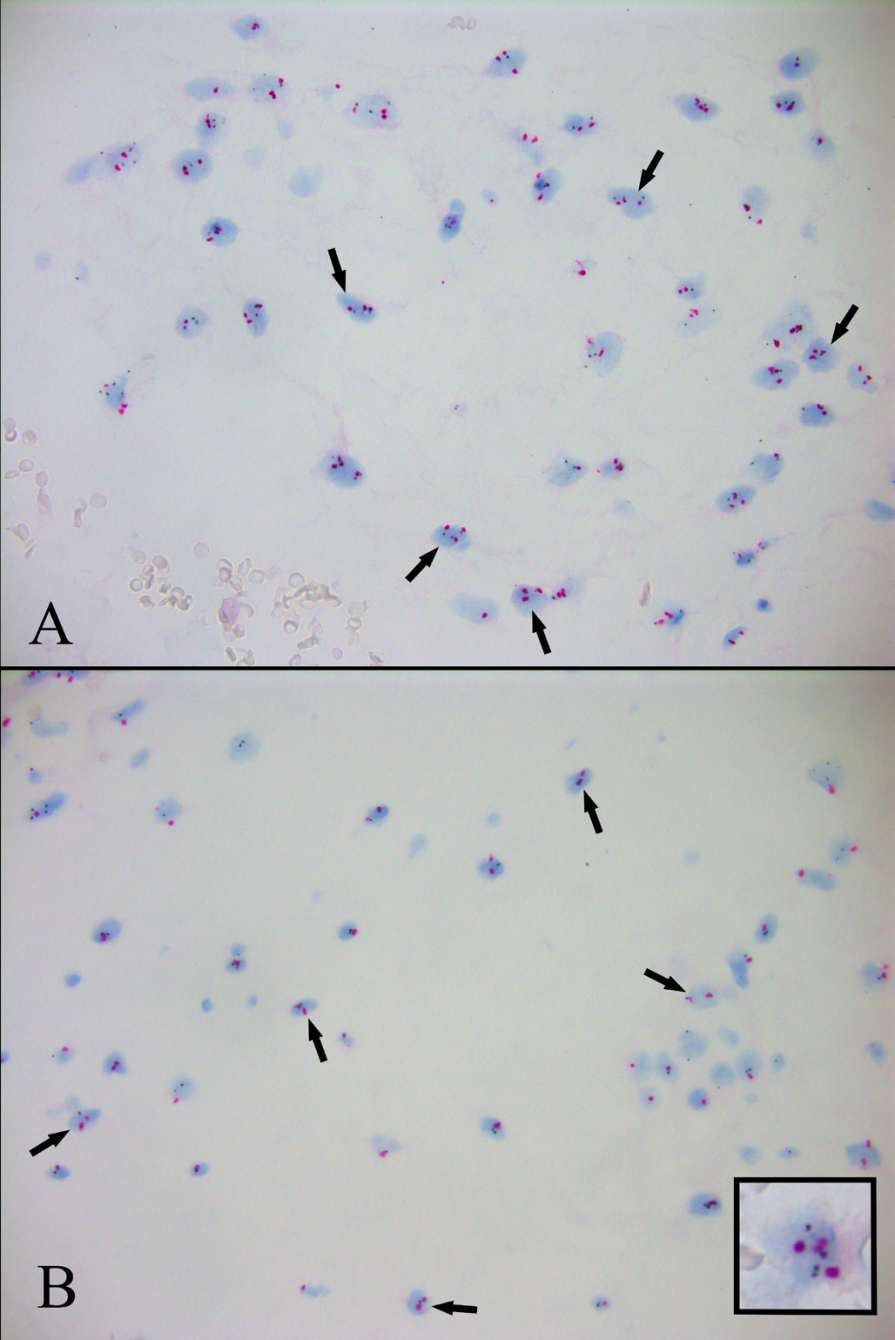
*HER2*/CEP17 D-DISH assay with black arrows showing (A) three red signals in some but not all villous stromal nuclei in a triploid conceptus and (B) two red signals in some but not all villous stromal nuclei in a diploid conceptus (nuclei with three signals are rare in this situation). In B, the inset shows an example of a nucleus with three red signals in a genetically confirmed diploid conceptus. As four black Her2 signals are present, this example likely represents two overlapping nuclei that could not be recognised as separate (these black signals although present are not used in the ploidy counting methodology). CEP17, chromosome 17 enumeration probe; D-DISH, dual-colour dual-hapten in-situ hybridisation.

When we initially adapted the *HER2* D-DISH method for counting the CEP17 signals, it became evident that a considerable number of nuclei within a field of view exhibited either zero or just one signal. This reduced the average nuclear signals in both diploid and triploid POCs, blurring the distinction between diploidy and triploidy. For instance, using this counting method, a triploid mole (figure 2A) with 54 nuclei (whole or partial) and 86 red signals resulted in an average nuclear signal of 1.59. In contrast, a diploid POC (figure 2B) with 53 nuclei (whole or partial) and 58 red signals resulted in an average nuclear signal of 0.91. This counting method did not intuitively reflect the number of genomes present. One would expect diploid (2n) and triploid (3n) conceptuses to yield scores closer to 2 and 3, respectively. As a result, we decided to establish our own scoring system.

**Figure 3:**
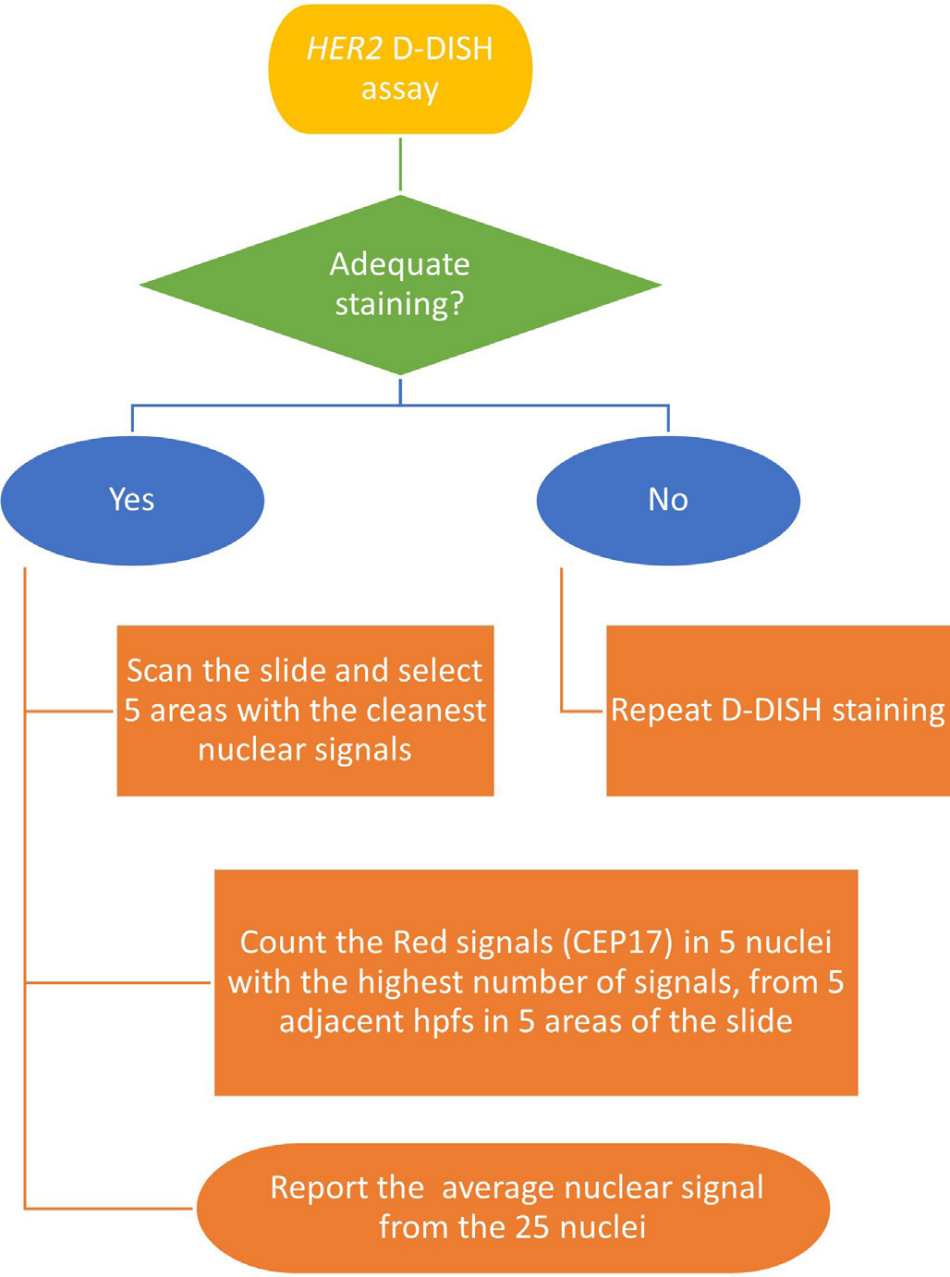
Flow diagram illustrating steps in the performance of the adapted *HER2* D-DISH assay for ploidy analysis and ‘rule of 5’ scoring system. CEP17, chromosome 17 enumeration probe; D-DISH, dual-colour dual-hapten in-situ hybridisation; hpfs, high-power fields.

We hypothesised that by focusing on a subset of nuclei with the highest nuclear signal counts within the high-power fields (hpfs) under evaluation, we would consistently identify and count nuclei with two CEP17 signals in diploid POCs and three CEP17 signals in triploid POCs. To validate this approach, we obtained the average of the highest nuclear signal count obtained from 5 nuclei, in 5 adjacent hpfs (x 400 magnification) in 5 different areas—the ‘rule of 5’ (figures3  4). To ensure this total number of nuclei was sufficient for scoring, we initially compared this method across 10 and 5 representative areas. Applying the basic principles of our newly established system to the *HER2* D-DISH example illustrated in figure 2, we found that a triploid mole (figure 2A) had five nuclei with three signals, resulting in an average nuclear signal of three. In contrast, a diploid POC (figure 2B) had five nuclei with two signals, resulting in an average nuclear signal of two. Hence, this scoring system aligns more intuitively with the evaluation of diploid and triploid status.

**Figure 4:**
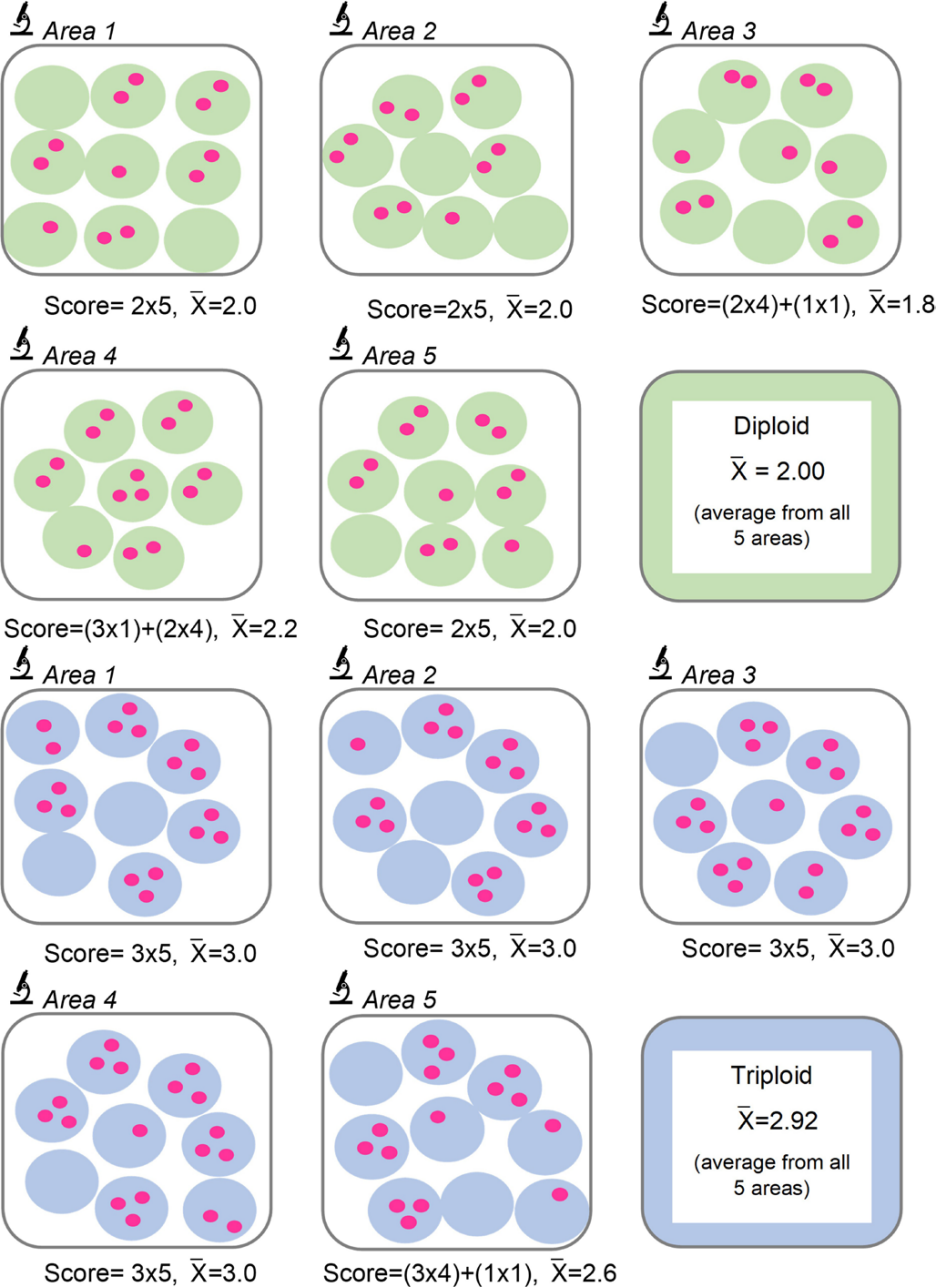
‘Rule of 5’ scoring system showing how the highest nuclear signals are selected. In each area, comprising 5 high-power fields (hpfs), the 5 nuclei with the highest number of chromosome 17 enumeration probe signals are identified and the average nuclear signal is recorded. This process is then repeated in 5 different areas of the slide to give an average across 25 nuclei. The average nuclear signal for diploidy is close to two and for triploidy is close to three. $\bar{X}$=average nuclear signal.

### Statistical analysis

The ‘rule of 3’ was used to estimate the power provided by our study sample size of 25 POCs. Applying this rule, we would have 95% confidence that the probability of an event occurring which is not seen in the validation cohort (ie, misclassification of diploidy or triploidy) is 12% (3 of 25), providing an estimated 88% power to determine study accuracy.^25^

The CEP17 nuclear signal counts were recorded in a scoring spreadsheet. Statistical analysis was performed using Microsoft Excel V.2021 and Analyse-IT software. Average nuclear signals were counted in 10 representative areas on each slide. The total scores assigned by three independent observers were compared using analysis of variance (ANOVA). The kappa statistic was used to assess inter-rater agreement across the three raters.

To determine the minimum number of areas required for scoring, a comparison was made between the average nuclear signal counts obtained from counting 5 vs 10 representative areas. Normality was assessed using the Shapiro-Wilk test. Parametric data were represented by mean (SD) and non-parametric data by median (IQR) and a p value of <0.05 was deemed statistically significant.

### Ploidy implementation audit

Application of the diagnostic threshold values established for ploidy status was audited for a 2-year period after implementation to assess its impact on diagnostics, its ease of implementation, clinical practice points and to determine the equivocal rates for PHM diagnoses.

## Results

In this study, the 25 cases selected had prior ploidy status established by molecular cytogenetics which reported 17 cases of diploidy (15 non-molar and 2 CHM) and 8 cases of triploidy (PHM).

Using our *HER2* D-DISH ploidy scoring system, we demonstrated complete concordance with the original genetic diagnosis. We successfully identified the correct number of diploid and triploid conceptuses yielding a 95% CI for a maximum sensitivity of ≥88%. Notably, the two diploid CHM cases identified by genetic analysis were diagnosed in our laboratory through morphological examination and ancillary p57 immunohistochemistry.

There was robust interobserver agreement between the three independent raters: A (mean 2.27, SD 0.45), B (mean 2.28, SD 0.47) and C (mean 2.25, SD 0.48), as assessed by ANOVA (p=0.36). The inter-rater agreement across the three raters, as measured by the kappa statistic, was 0.812 (p<0.001). If we exclude the two cases ultimately categorised as equivocal due to suboptimal staining, the kappa statistic is 0.927 (p<0.001). In keeping with Landis and Koch,^26^ kappa values of 0.81 or more may be interpreted as almost perfect agreement.^26^ This highlights the need for good-quality staining to ensure more accurate counts and better precision when applying this novel scoring system.

Average nuclear signal counts for diploid and triploid cases remained constant when we reduced the count from 10 to 5 representative areas, as depicted in table 1. This consistency held true when we analysed all cases collectively, and when we excluded the two equivocal cases from our analysis. Consequently, we can confidently adopt a scoring system that relies on just five areas characterised by high signal intensity. This finding emphasises the reliability and robustness of our scoring system.

**Table 1:** Average nuclear signal counts from 5 vs 10 representative areas

Cases	x̄_5_	SD	x̄_10_	SD	P value
Total (n=25)	2.27	0.39	2.26	0.39	0.4524
Total (n=23)*	2.27	0.40	2.26	0.40	0.3048
Diploid (n=17)	2.03	0.05	2.04	0.06	0.2334
Triploid (n=8)	2.78	0.28	2.77	0.26	0.9453

x̄_5_=mean average nuclear signal count for 5 areas on each slide; x̄_10_=mean average nuclear signal count for 10 areas on each slide.

P value: statistical significance (p<0.05).

* Equivocal cases excluded,.($\bar{X}$) mean average nuclear signal count for 5 areas on each slide ($\bar{X}$) average nuclear signal count for 10 areas on each slide, : standard deviation. P value: statistical significance (p).

### Establishing decision thresholds

**Figure 5:**
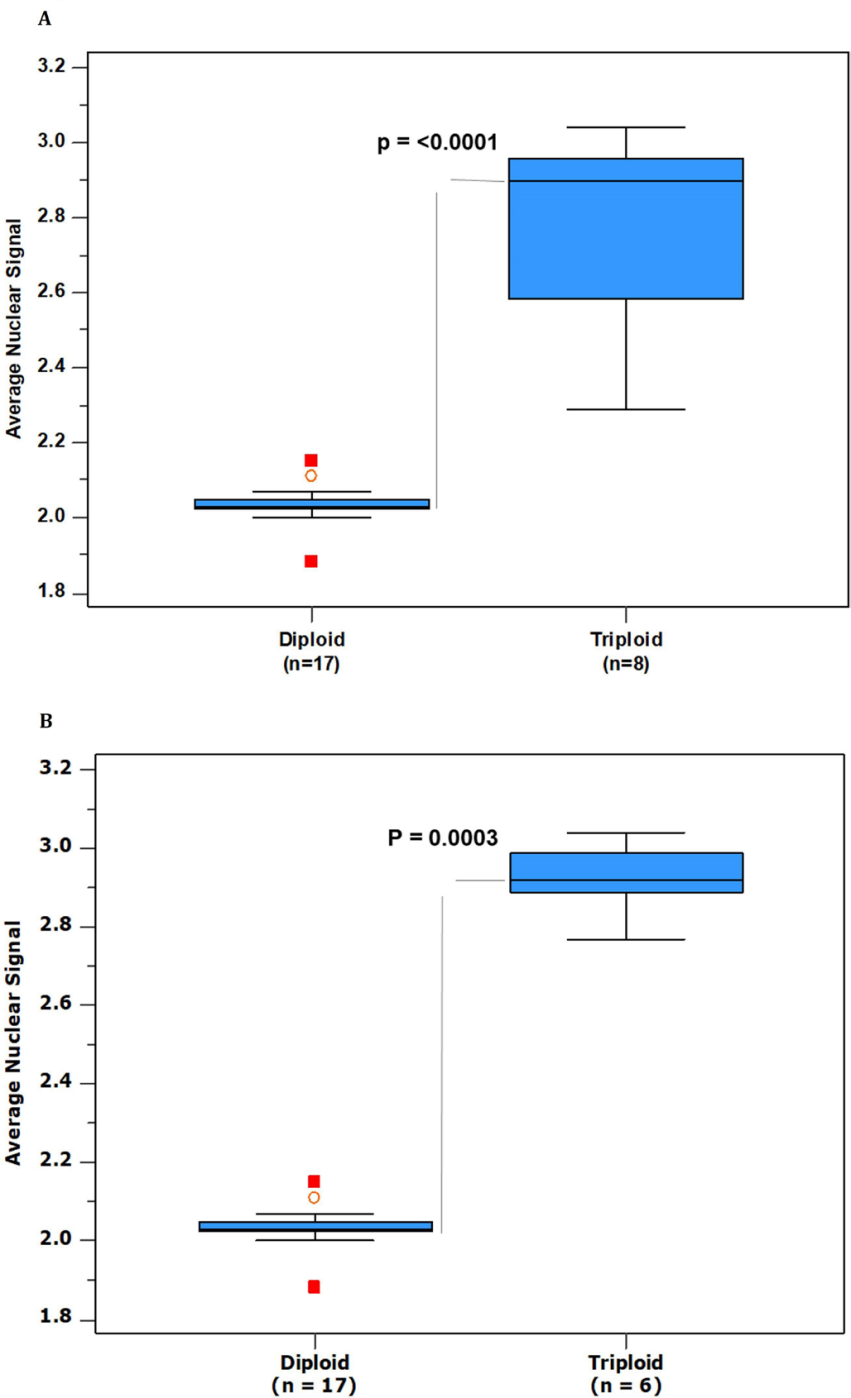
Box and whisker plots showing the average nuclear signal based on the ‘rule of 5’ scoring system. Scores are divided into diploid and triploid conceptuses to enable the establishment of ploidy decision thresholds. (A) All 25 cases and (B) 23 cases (equivocal cases excluded). The box portion of the plot includes 50% of the data, the lower, median (represented by a solid line) and upper quartile. The whiskers extend to the maximum and minimum values. Disconnected points are potential outliers. Grey lines show statistical significance (p<0.05) between diploid and triploid medians.

Ploidy decision cut-off values were determined by considering all 25 cases collectively and then re-evaluating with the exclusion of the 2 cases ultimately deemed ‘equivocal’. Notably, there was a statistically significant separation between the average nuclear signal counts for diploid and triploid conceptuses, as demonstrated using the Wilcoxon test (p<0.05). This held true even when equivocal cases were included and validated the application of our innovative ‘rule of 5’ scoring system (figure 5).

The distinction between diploid and triploid conceptuses is visually depicted using box and whisker plots, illustrating the average nuclear signal counts for both groups (figure 5). Final decision thresholds were established based on the analysis of 23 cases (table 2). A conceptus was categorised as diploid (2n) if it exhibited an average nuclear signal ≤2.2 and triploid (3n) if it displayed an average nuclear signal of ≥2.8. Cases falling within the range of scores >2.2 and <2.8 were categorised as equivocal.

**Table 2:** Ploidy decision thresholds for diploid and triploid genomes

Ploidy status	Genome number (n)	‘Rule of 5’-based average nuclear signal
Diploid	2n	≤2.2
Equivocal	2n–3n	>2.2 and <2.8
Triploid	3n	≥2.8

It is worth noting that two cases ultimately designated as having ‘equivocal’ scores (cases 14 and 19) were indeed triploid but displayed scores slightly lower than the other triploid conceptuses, with scores of 2.29 and 2.40, respectively. Upon review, it became apparent that both cases had suboptimal staining quality, which likely accounted for the lower average nuclear signals observed. Importantly, neither of these cases exhibited characteristic morphological features of PHM and required ploidy analysis to establish the diagnosis. While their scores clearly distinguished them from the diploid cases (all of which had scores less than 2.15), it was decided to place these two cases in an equivocal category to maximise diagnostic confidence. This approach ensured a substantial gap between diploid and triploid categories (figure 5B).

### Implementation audit results

Following the implementation of our ploidy determination method with established decision thresholds, an audit of all POCs received in our pathology department over a 2-year period revealed that 7.7% (98 of 1264) of all POCs underwent *HER2* D-DISH analysis to aid in the diagnosis or exclusion of PHM. Among those cases, 47% (46 of 98) were confirmed as triploid PHMs, with an equivocal rate of 1.02% (1 of 98). In collaboration with clinicians on our national GTD steering committee, it was decided that all equivocal cases would be registered with the national GTD centre for further clinical follow-up and hCG monitoring as potential PHMs. This decision was made due to the lack of molecular genotyping services nationally, which could have resolved the equivocal diagnoses, and the short surveillance period required for most PHMs.

During the practical implementation of this method, pathologists found that the scanning process, aimed at identifying nuclei with the highest signal count in each hpf, effectively evolved into a search for nuclei with three or more signals. This approach allowed for the rapid evaluation of a substantial number of nuclei within the villus stromal population, spanning a total of 25 hpfs. As a result, it enabled a more comprehensive assessment of the tissue than initially appreciated.

## Discussion

Our validation study has demonstrated the accuracy of *HER2* D-DISH ploidy analysis in effectively distinguishing between diploid and triploid gestations. While the initial validation study identified 2 out of 25 cases (8%) as equivocal, continuous experience gained in morphological assessment and *HER2* D-DISH staining has substantially reduced the occurrence of equivocal cases over time. This reduction is exemplified by the lower equivocal rate (1.02%) observed in our implementation audit of the ‘rule of 5’ scoring system, confirming the diagnostic utility of this technique.

We established the sensitivity and specificity of our novel scoring system in a follow-up evaluation audit using molecular genotyping. This audit aimed to assess the accuracy of the *HER2* D-DISH ploidy assay in identifying triploid PHM conceptuses (sensitivity) and diploid non-molar conceptuses (specificity) in a cohort of samples with equal numbers of diploid and triploid conceptuses. Application of the scoring system to determine ploidy did not yield any false positive or false negative triploid PHM results. This audit achieved 100% sensitivity and specificity (95% CI: ≥92%, n=36) confirming the accuracy of our novel scoring system.^22^

It is important to note that ploidy analysis, while valuable in distinguishing diploid from triploid conceptuses, does not identify the genomic origin of the additional chromosomal complement in a triploid conceptus. This raises concerns about the potential overdiagnosis of PHM based on ploidy alone, as there is a risk of mistakenly including digynic triploid conceptuses. Additionally, there are documented cases where digynic triploid non-molar pregnancies exhibit morphological features similar to PHM.^9^ However, recent auditing of our service using molecular genotyping demonstrated that *HER2* D-DISH ploidy analysis did not lead to misdiagnosing PHM due to digynic triploidy.^22^ This suggests that, from a practical viewpoint, laboratories using morphological assessment supported by an accessible ploidy assay may (a) accurately exclude PHM by confirming diploid conceptus status and (b) confirm triploid status in suspected PHM cases without a substantial risk of overdiagnosis by miscategorising digynic triploid conceptuses.

Use of molecular genotyping for ploidy analysis has certain challenges, primarily due to the low quantity and quality of DNA extracted from FFPE tissue, which in turn, impacts the success rate of the analysis. The potential for contamination of trophoblastic villi with maternal decidua further complicates the interpretation of genotyping results. Furthermore, it is important to recognise that genotyping represents a more expensive approach when compared with *HER2* D-DISH, principally due to the requirement for specialist equipment and the need for scientists proficient in interpreting genotyping data.

In our laboratory, we have a low threshold for requesting ploidy analysis to support PHM diagnosis. The key enabler of this approach is the ready availability of *HER2* D-DISH analysis, which has proven to be highly effective in our setting. We have successfully identified triploidy in approximately 47% of atypical conceptuses selected for testing, coupled with a low equivocal rate of 1.02%. The adapted *HER2* D-DISH assay offers many advantages, most notably its speed, convenience, ready accessibility and compatibility with FFPE tissue. It has many benefits over molecular genotyping including the potential for a quicker turnaround time, broader accessibility within pathology laboratories and the absence of a requirement for specialist training in molecular genetics.

In laboratories that lack access to ancillary techniques for aiding PHM diagnosis, especially in cases where a definitive diagnosis cannot be reached and PHM cannot be excluded, adherence to national clinical guidelines is advisable. In Ireland, these guidelines recommend registering such cases with the national GTD centre for hCG surveillance, as hCG levels generally normalise within a 2-month period.^27^

The finding that a relatively high proportion (7.7%) of POCs received by our laboratory required the application of *HER2* D-DISH ploidy analysis to aid in PHM diagnosis or exclusion is noteworthy. Equally striking is the almost equal distribution between the identification of triploid (47%) and diploid (52%) cases. This collectively underscores the complexity of HM diagnosis, emphasising the importance of having an accessible solution to address these challenges in clinical practice.

### Limitations and strengths

Future prospective larger studies are needed to validate this innovative scoring system before it can be recommended for routine use and especially as it extends to other pathology centres. Given the broader availability of in-situ hybridisation in pathology laboratories, this makes the integration of in-situ hybridisation into routine practice more feasible than molecular genotyping. Going forward, implementation of *HER2* D-DISH ploidy analysis holds the promise of reducing the number of cases with uncertain diagnoses, thereby alleviating emotional distress for patients and eliminating the costs associated with clinic attendance and hCG surveillance. Moreover, it has the potential to address an unmet clinical need by providing more accurate estimates of HM incidence rates.

## Conclusion

Use of the adapted *HER2* D-DISH assay, using the innovative ‘rule of 5’ scoring system, provides a reliable adjunct to morphological assessment for partial hydatidiform mole diagnosis. This ploidy assay offers a potentially more accessible alternative to molecular genotyping, particularly in pathology laboratories where resources are limited.

## Data Availability

All data relevant to the study are included in the article or uploaded as supplemental information.
